# Microtensiometers Accurately Measure Stem Water Potential in Woody Perennials

**DOI:** 10.3390/plants10122780

**Published:** 2021-12-16

**Authors:** Victor Blanco, Lee Kalcsits

**Affiliations:** 1 Tree Fruit Research and Extension Center, Washington State University, Wenatchee, WA 98801, USA; victor.blanco@wsu.edu; 2 Department of Horticulture, Washington State University, Pullman, WA 99164, USA

**Keywords:** gas exchange, irrigation scheduling, pear, precision agriculture, pressure chamber, sensors, tree water status, vapor pressure deficit, water stress indicators

## Abstract

Stem water potential (Ψ_stem_) is considered to be the standard measure of plant water status. However, it is measured with the pressure chamber (PC), an equipment that can neither provide continuous information nor be automated, limiting its use. Recent developments of microtensiometers (MT; FloraPulse sensors), which can continuously measure water tension in woody tissue of the trunk of the tree, can potentially highlight the dynamic nature of plant water relations. Thus, this study aimed to validate and assess the usefulness of the MT by comparing the Ψ_stem_ provided by MT with those same measurements from the PC. Here, two irrigation treatments (a control and a deficit treatment) were applied in a pear (*Pyrus communis* L.) orchard in Washington State (USA) to capture the full range of water potentials in this environment. Discrete measurements of leaf gas exchange, canopy temperature and Ψ_stem_ measured with PC and MT were made every two hours for four days from dawn to sunset. There were strong linear relationships between the Ψ_stem_-MT and Ψ_stem_-PC (R^2^ > 0.8) and with vapor pressure deficit (R^2^ > 0.7). However, Ψ_stem_-MT was more variable and lower than Ψ_stem_-PC when Ψ_stem_-MT was below −1.5 MPa, especially during the evening. Minimum Ψ_stem_-MT occurred later in the afternoon compared to Ψ_stem_-PC. Ψ_stem_ showed similar sensitivity and coefficients of variation for both PC and MT acquired data. Overall, the promising results achieved indicated the potential for MT to be used to continuously assess tree water status.

## 1. Introduction

Precision agriculture technologies are necessary to improve labor and natural resource-use efficiency. Many tree-fruit-producing regions require irrigation water to be profitable. [1]. Advances in soil water-based irrigation scheduling have improved irrigation scheduling based on climatic conditions, reference evapotranspiration, and crop coefficients [2,3]. However, there is uncertainty about making irrigation decisions without directly measuring the tree, considering temporal and spatial variability in weather and soil present in agricultural systems. Consequently, the use of plant-based indicators that directly measure tree water status represents a significant step towards precision irrigation to avoid undesirable water stress, which may penalize fruit quality and yield, but also overwatering which can waste limited resources and soil nutrients while increasing costs [4]. Among all the plant-based water status indicators, stem water potential (Ψ_stem_) is considered one of the most accurate plant-based water status measures for fruit trees and vines [5]. Stem water potential is the direct measure of the tree water status by measuring the water tension within the plant (MPa). It is traditionally used as the reference to compare against other water status indicators [6]. Currently, the main limitation of Ψ_stem_ is the time required to make measurements. To measure Ψ_stem_, a healthy, non-sun-exposed leaf located close to the trunk needs to be covered with black polyethylene plastic and aluminum foil for two hours to limit transpiration to allow leaf water potential to reach an equilibrium with Ψ_stem_ [7]. It is a temporally discrete, labor-demanding, destructive measurement and requires a Scholander pressure chamber (PC) [8]. The pressure chamber measures the water potential by applying pressurized gas to a chamber where the excised leaf is placed with the petiole extending outside the sealed chamber [9]. The chamber measures the amount of pressure required to force sap to pool at the end of the petiole. Higher pressure requirements inside the chamber (e.g., 1.5 MPa) indicate that water inside the plant tissue is strongly retained, so the water potential of the plant is lower [10]. Low Ψ_stem_ values are related to low sap-flow velocities and can be caused by low soil moisture [11]. Thus, soil water deficit, as well as other environmental factors, has been reported to affect the development of vascular tissues such as the xylem vessels of the tree, decreasing its density, which modifies tree water movement through the trunk and decreases stem water potential [12].

Knowing that the pressure chamber cannot continuously measure Ψ_stem_, and with the aim of continuously monitoring tree water status, other continuous plant-based indicators such as those derived from the trunk diameter fluctuations [13], sap flow [14], leaf turgor pressure [15], or canopy thermal index [16] have been developed. However, despite the development of these continuous measures, commercial implementation has been limited, mainly by their price and the difficult process of data interpretation. Other sensors have been more recently developed such as time and frequency domain reflectometry sensors that measure tree stem water content [17], but their use is even more limited and they have not been yet compared to other commonly used water status indicators such as Ψ_stem_.

In this sense, microtensiometers (MT) appear as an option for continuous monitoring of water status. Microtensiometers measure water potential based on a microelectromechanical pressure sensor that can do in situ measurements [18]. They are embedded in the trunk and directly measure stem water potential. This is a promising method for stem water potential determination, as it can be automated providing continuous data in easy-to-interpret pressure units such as traditional pressure chamber stem water potential methods. However, there is no information available comparing the stem water potential measurements of the microtensiometers with other well-known tree water status indicators under field conditions and how it relates to functional physiology measures for woody perennial trees. In this sense, the scarce information currently available on microtensiometers is focused on vines, and for Ψ_stem_ values higher than −1.0 MPa [19].

We hypothesized that microtensiometers can provide sensitive, stable, and accurate stem water potential measurements, and we sought to know if they can be used within a wide range of environmental and soil water conditions. Thus, the aim of this study was to validate the use of microtensiometers as plant-based water status sensors and compare stem water potential values acquired from microtensiometers with those values measured with the pressure chamber across different irrigation strategies, times throughout the day, and days with different environmental conditions. To our knowledge, this is the first report that shows MT response to different environmental and soil water content situations and compares these results with those taken by traditional discrete plant-based water status indicators in mature, field-grown pear trees.

## 2. Results

### 2.1. Weather and Soil Conditions

**Figure 1:**
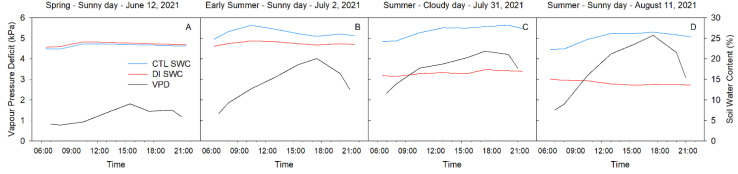
Diurnal patterns of mean volumetric soil water content (SWC) and vapor pressure deficit (VPD) for four representative days (12 June (**A**), 2 July (**B**), 31 July (**C**), 11 August (**D**)) during 2021 season.

The meteorological conditions, as well as the soil water content, were different each selected day. However, overall, the daily pattern of variables such as air temperature and vapor pressure deficit (VPD) followed a similar trend with daily maximum and minimum values during the afternoon (16:00 h) and the sunrise (06:00 h), respectively. Reference evapotranspiration was lowest on 12 June 2021 (ET_0_ = 6.02 mm) and was characterized by mean air temperature and VPD values of 19.3 °C and 1.34 kPa (Figure 1A). Atmospheric demand was higher for the other three days, and the mean air temperature and VPD were 27.5 °C and 2.68 kPa on 2 July (ET_0_ = 10.16 mm; Figure 1B), 31.2 °C and 3.23 kPa on 31 July (ET_0_ = 7.62 mm; Figure 1C), and 30.6 °C and 3.25 kPa on 11 August (ET_0_ = 8.13 mm; Figure 1D). These days represent a range in environmental conditions present during the summer in a hot, semi-arid environment.

Photosynthetically active radiation (PAR) was similar for the three sunny days (12 June, 2 July and 11 August) with maximum values above 1500 µmol m^−2^ s^−1^. They were all greater than the cloudy day (31 July) when the maximum PAR values were below 900 µmol m^−2^ s^−1^.

Soil water content (SWC) was strongly influenced by the irrigation strategy applied. On 12 June, both treatments (CTL and DI) were equally irrigated to satisfy tree water requirements (100% ET_c_), so consequently, both treatments were the same (Figure 1A). However, from June 28^th^ onwards, trees from the DI treatment were irrigated to satisfy 50% of the ET_c_. As such, there were strong differences in soil water content between treatments (Figure 1B–D). SWC was similar for all the selected days in the CTL treatment with daily mean values that ranged between 23 and 28%. However, daily mean SWC values for DI trees were 23% on 2 July, 16% on 31 July, and 14% on 11 August, which showed the progressive depletion of soil water content under trees where DI was applied.

### 2.2. Stem Water Potential. Diurnal Pattern

**Figure 2:**
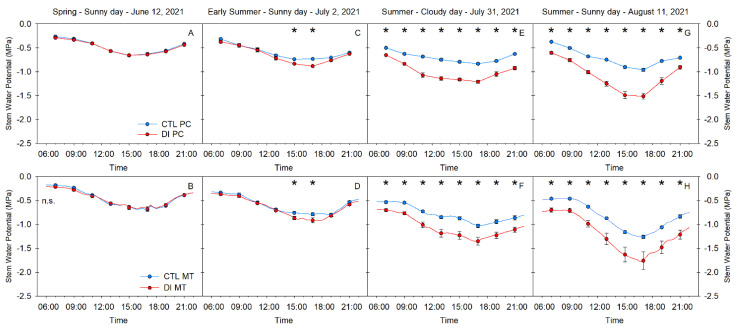
Diurnal patterns of stem water potential measured with the pressure chamber (PC) (**A**,**C**,**E**,**G**) or with the microtensiometer (MT) (**B**,**D**,**F**,**H**) for control (CTL, blue) and deficit-irrigated (DI, red) trees on four representative days (12 June, 2 July, 31 July, 11 August) during the 2021 growing season (N = 6). Asterisks indicate statistically significant differences between irrigation treatments according to ANOVA (*p* ≤ 0.05).

Ψ_stem_ measured by both the pressure chamber and the microtensiometers was similarly diurnal for all four days of study and was clearly influenced by the environment. Ψ_stem_ was correlated with VPD. As evaporative demand increased, Ψ_stem_ decreased, reaching the daily minimum value in the early afternoon, after midday, when the VPD reached its maximum value. Once VPD values gradually decreased during the evening, Ψ_stem_ continued to recover (Figure 2). Ψ_stem_ for CTL trees was highly dependent on atmospheric demand rather than soil moisture. For example, on days with VPD values higher than 5 kPa, Ψ_stem_ for CTL trees was greater than −0.90 MPa measured with the pressure chamber and higher than −1.20 MPa when they were measured with microtensiometers (Figure 2G,H).

On the other hand, Ψ_stem_ for trees with DI applied, which were under soil water restrictions during the summer (2 July, 31 July, and 11 August), showed minimum values which became progressively more negative during the season due to the combination of high evaporative demand and low water availability in the soil. Daily minimum Ψ_stem_ for trees with DI applied decreased from −0.8 MPa on 2 July (Figure 2C,D) to values ranging between −1.5 and −2.0 MPa on 11 August (Figure 2G,H). Similarly, maximum daily Ψ_stem_ for trees with DI applied gradually decreased from −0.35 MPa (2 July) to −0.65 MPa (11 August), which indicated that these trees were water-stressed and water status was not able to fully recover during the night.

Ψ_stem_ was a sensitive tree water status indicator that was able to distinguish between irrigation treatments. Ψ_stem_ was significantly different between irrigation treatments for all the measurements taken on 31 July and 11 August (Figure 2E–H). However, on 2 July, only the early afternoon stem water potential was significantly different between irrigation treatments five days after imposing the deficit irrigation (Figure 2C,D). Both methods of measuring Ψ_stem_ were able to differentiate between the two irrigation treatments. Moreover, they showed significant differences on the same day and at the same time, further supporting the agreement between both methods.

### 2.3. Stem Water Potential. Comparative Response: Pressure Chamber vs. Microtensiometers

**Figure 3:**
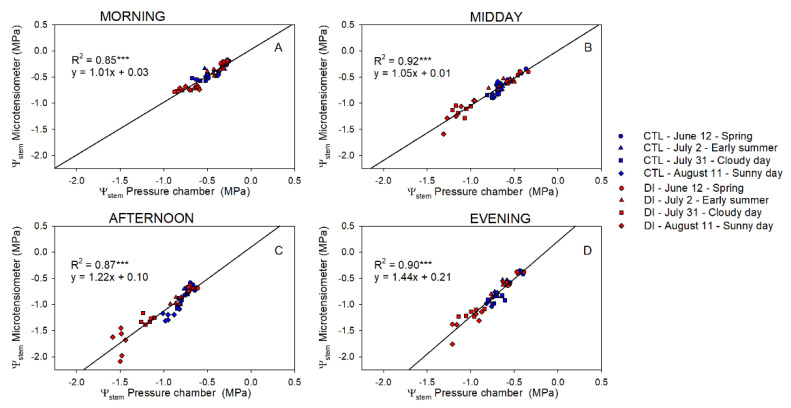
Relationship between stem water potential measured with the pressure chamber and the microtensiometers for control trees (CTL, blue) and deficit irrigated trees (DI, red) in the morning (**A**), midday (**B**), afternoon (**C**), and evening (**D**) of four representative days (12 June (circle), 2 July (square), 31 July (triangle), 11 August (diamond)) during 2021 season. (*** *p* < 0.001).

There was a strong linear relationship between the Ψ_stem_ measured with PC and MT (*p*-value < 0.001; R^2^ ≈ 0.89; Figure 3). However, the linear relationship between them became divergent from the one-to-one line (y = x), highlighting the differences between both methods at lower Ψ_stem_ values.

**Figure 4:**
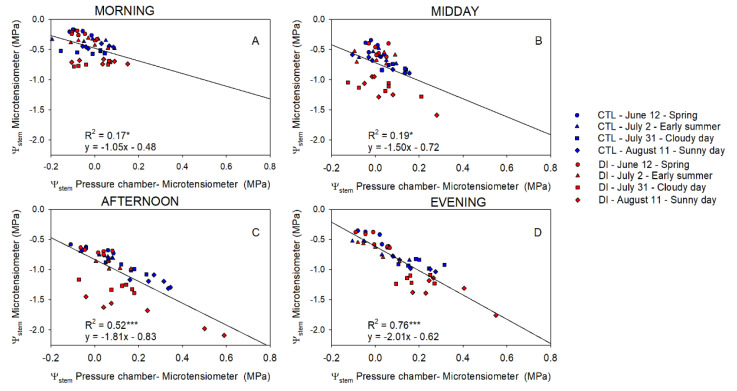
The linear relationship between stem water potential measured with the microtensiometers and the difference between the pressure chamber and the microtensiometers for control trees (CTL, blue) and deficit irrigated trees (DI, red) in the morning (**A**), midday (**B**), afternoon (**C**), and evening (**D**) of four representative days (12 June (circle), 2 July (square), 31 July (triangle), 11 August (diamond)) during 2021 season. (* *p* < 0.05, *** *p* < 0.001).

**Figure 5:**
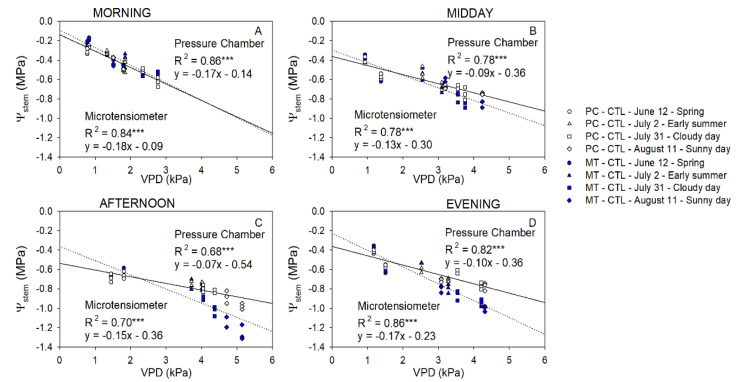
Relationship between the stem water potential measured with the microtensiometers (MT, blue) and the pressure chamber (PC, white) with the vapor pressure deficit (VPD) for control trees (CTL) in the morning (**A**), midday (**B**), afternoon (**C**), and evening (**D**) of four representative days (12 June (circle), 2 July (square), 31 July (triangle), 11 August (diamond)) during 2021 season. (*** *p* < 0.001).

Differences between Ψ_stem_ of trees using either CTL or DI strategies ranged between +0.1 and −0.3 MPa in the morning. Differences between the two irrigation treatments were greater when MT were used to measure Ψ_stem_. The greatest differences between the pressure chamber and the microtensiometers were observed when values were the lowest regardless of the time of the day (Figure 4). Ideally, the coefficient of determination between Ψ_stemPC-MT_ and Ψ_stem_ measured with microtensiometers should be close to zero. However, that was not the case in this experiment. Microtensiometers consistently underestimated Ψ_stem_ values below −1.5 MPa, reaching values between 0.3 and 0.6 MPa below those measured with the pressure chamber, as can be seen in DI trees on 11 August, afternoon and evening (Figure 4C,D). Moreover, the influence of the evaporative demand on Ψ_stem_ measured with PC and MT was assessed. Both methods showed a strong relationship between Ψ_stem_ and VPD (Figure 5).

Ψ_stem_ was the most linear and comparable between methods in the morning. The relationship between Ψ_stem_ for both methods and VPD started to diverge when VPD decreased below 3 kPa. This trend was also exhibited in the afternoon and in the evening, indicating that Ψ_stem_ measured with MT might be more sensitive to evaporative demand (Figure 5).

### 2.4. Stem Water Potential. Level of Tree Water Stress

**Figure 6:**
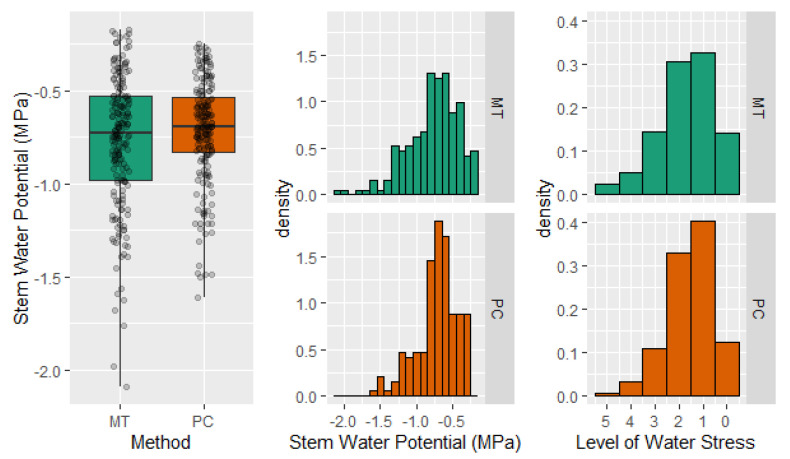
Stem water potential mean value and distribution according to a 0.1 MPa scale and a water stress scale (0 absence of water stress, 5 severe water stress) for stem water potential values measured during the experiment with the pressure chamber (PC) and the microtensiometers (MT).

Pooled data indicated that microtensiometers resulted in a greater dispersion of Ψ_stem_, with values that ranged between −0.2 and −2.1 MPa, while Ψ_stem_ measured with the pressure chamber ranged between −0.3 and −1.6 MPa (Figure 6). Similar results were observed when the frequency distribution of measured Ψ_stem_ values was calculated for each method. However, when the Ψ_stem_ values were translated into values according to a scale of water stress, those differences in the distribution of the data decreased. This new distribution exhibited that the differences in Ψ_stem_ between methods generally did not imply a different level of water stress for the tree (Figure 6). These results highlight the applicability of both methods and relativize the differences found between them.

### 2.5. Gas Exchange and Canopy Temperature. Diurnal Pattern

Leaf gas exchange (stomatal conductance (*G*_s_) and net photosynthesis (*P*_n_)) increased from 08:00 h to 10:30 h (the period of maximum stomatal conductance and carbon assimilation), from 10:30 h to midday; *G*_s_ was mostly stable or still increasing at a slow rate while *P*_n_ decreased. During the afternoon, leaf gas exchange continued to decrease reaching the daily minimum value in the evening.

**Figure 7:**
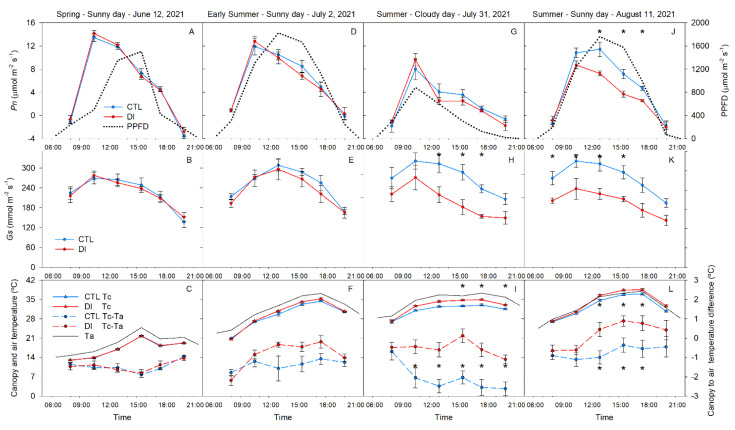
Diurnal course of photosynthetic photon flux density (PPFD), net photosynthesis (*P_n_*) (**A**,**D**,**G**,**J**), stomatal conductance (*G_s_*) (**B**,**E**,**H**,**K**), canopy temperature (Tc), air temperature (Ta), and the difference between Tc and Ta (Tc-Ta) (**C**,**F**,**I**,**L**) for control (CTL, blue) trees and deficit-irrigated (DI, red) trees on four representative days (12 June, 2 July, 31 July, 11 August) during the 2021 season (N = 6). Asterisks indicate statistically significant differences between irrigation treatments according to ANOVA (*p* ≤ 0.05).

There were no differences in *G*_s_ or *P*_n_ between irrigation treatments on 12 June or 2 July (Figure 7). On 31 July, the cloudy day, *P*_n_ values were similar for both treatments and were negatively affected by the lack of solar radiation. *P*_n_ was 21 and 35% lower for the cloudy day compared to measurements made on the sunny day for the CTL and DI trees, respectively (Figure 7G,J). CTL trees had significantly greater *P*_n_ than trees with DI applied. Similarly, *G*_s_ of DI trees was lower for trees where DI was applied compared to the control trees. These differences were the greatest on 11 August, the day with the highest temperature and solar radiation (Figure 7B,E,H,K). On 31 July, although the trees from both treatments maximum *G*_s_ by 10:30 h, *G*_s_ rapidly decreased in DI trees, while CTL trees decreased at a slower rate (Figure 7).

Canopy temperature (T_c_) was heavily influenced by ambient air temperature and the irrigation strategy and was similar to gas exchange measurements. Maximum canopy temperature was observed during the afternoon, 3 h after maximum *G* was observed. However, like *P_n_* and *G*, there were no differences in T_c_ between treatments in the morning (Figure 7L). On the other hand, when the difference between the air (T_a_) and the canopy temperature was considered (T_c_-T_a_), differences appeared earlier at midday (Figure 7I). On the hottest sunny day (11 August), maximum difference between canopy and air temperature (1 °C) occurred at 15:30 in DI trees.

### 2.6. Stem Water Potential Relationship with Stomatal Conductance and Canopy Temperature

**Figure 8:**
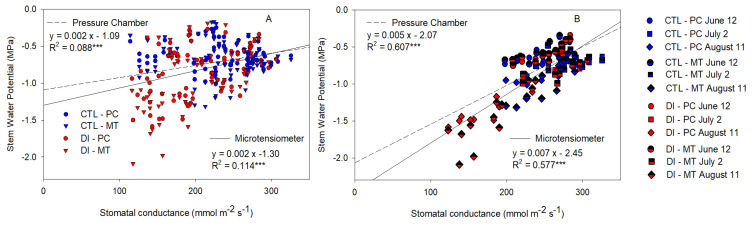
Relationship between the stem water potential measured with the microtensiometers (MT) and the pressure chamber (PC) with the stomatal conductance for control trees (CTL, blue) and deficit-irrigated trees (DI, red) of four representative days (12 June (circle), 2 July (square), 31 July (triangle), 11 August (diamond)) (**A**) and three representative days (12 June (circle), 2 July (square), 11 August (diamond)) from 10:30 to 15:30 h during 2021 season (**B**). (*** *p* < 0.001).

The linear relationships between *G_s_* and Ψ_stem_ measured with PC and MT were calculated for all the data (Figure 8A) and when only the data measured between 10:30 and 17:30 h on sunny days were considered (Figure 8B). As expected, the relationship was stronger when only the sunny days were considered. Both methods showed similar and significant relationships with *G_s_*, PC had a greater coefficient of determination when all the values were considered, while MT had a slightly stronger relationship when only the sunny days were taken into account.

**Figure 9:**
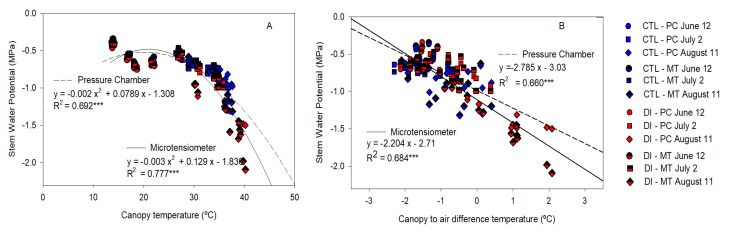
Relationship between stem water potential measured with the microtensiometers (MT) and the pressure chamber (PC), and canopy temperature (**A**) and canopy-to-air temperature (**B**) for control trees (CTL, blue) and deficit-irrigated trees (DI, red) of three representative days (12 June (circle), 2 July (square), 11 August (diamond)) from 10:30 to 15:30 h during 2021 season. (*** *p* < 0.001).

In the same vein, canopy temperature resulted strongly related to Ψ_stem_ measured by both PC and MT, when the data from 10:30 to 17:30 h of the sunny days were considered (Figure 9A); however, the relationship calculated was not suitable for those Ψ_stem_ values higher than −1.0 MPa. On the other hand, when the difference between the canopy and the air temperature was considered, the linear relationship had a better match with the most negative values (Figure 9B).

### 2.7. Sensitivity Analysis

Ψ_stem_ was clearly the plant-based water status indicator with the highest signal intensity (SI) and sensitivity (S) followed by *G_s_* and T_c_-T_a_. Regarding Ψ_stem_, the mean value of SI for both methods was similar across the day (1.25, Table 1), with differences not exceeding 5% of variability. However, when different days of the season were compared, SI increased as the season progressed from SI values of 1 to 1.5 (Table 2). Furthermore, SI was similar between PC and MT for all days (Table 2).

The coefficient of variation (CV) was slightly lower for PC (0.05) than for MT (0.07). Sensitivity (S) was high for both methods, and S was not below 12 for any of the time periods. When S was compared for each day, S was 24 and 37% higher for PC than for MT. Although both methods were sensitive to changes in tree water status, S was particularly higher for PC in the afternoon, but also in the morning and in the evening, while the microtensiometers were slightly more sensitive at midday.

## 3. Discussion

Ψ_stem_ remains one of the most important indicators of tree water status. It integrates soil, plant, and atmospheric factors and was stable for both methods of measurement in this experiment. It was able to distinguish between irrigation treatments and was sensitive to changes in evaporative demand and radiation throughout the day. Both methods, the PC and the MT, supplied similar Ψ_stem_ data (R^2^ > 0.8; Figure 3) when compared at the same time. However, PC measurements are time-point measurements, and MT provide continuous data. Ψ_stem_ is widely regarded as one of the most useful plant water indicators for trees [6,20,21]. However, since Ψ_stem_ have traditionally been discrete data, there has been extensive research to identify both direct and indirect assessments of plant water status. Thus, several attempts were made considering easily measured environmental variables such as VPD or air temperature and soil water content/potential [8,22,23], as well as non-destructive, discrete [24,25,26], and continuous [13,27,28] plant-based sensors and high-resolution imagery [29,30,31]. Although these research papers provided interesting results that relate with Ψ_stem_, they also emphasized the necessity of validating their results under different soil and climate conditions as well as under different cultivars. In this sense, the use of Ψ_stem_ acquired through MT may be more useful since it is a direct assessment of plant water status.

Other measurements of water status were not as sensitive as Ψ_stem_ in this experiment. Ψ_stem_ was the first measure to detect water stress five days after water limitations were imposed. Gas exchange (*P_n_* and *G_s_*) and canopy temperature were not significantly different between treatments at that time and resulted in lower signal intensity and sensitivity (Table 2). However, for the two later dates (31 July and 11 August), water limitations affected *P_n_*, *G_s_*, and T_c_ (Figure 7). The delayed response of leaf gas exchange under water limitations compared to Ψ_stem_ has been widely reported [32]. These differences were also limited on 31 July (the cloudy day). Overall, T_c_ was the indicator with the lowest response, followed by *P_n_*, which on the cloudy day was unable to distinguish between CTL and DI trees despite the great difference in volumetric soil water content. Aside from Ψ_stem_, T_c_-T_a_ and *G_s_* consistently showed differences between irrigation treatments (Figure 7).

In this sense, Ψ_stem_ is sometimes not considered to be an early indicator of water stress [33,34]. Specifically, these experiments compared predawn or midday water potential during the first few days of water limitations. These results align with our observations where Ψ_stem_ was not able to distinguish between irrigation strategies at these times. However, significant differences between irrigation treatments appeared later in the afternoon (Figure 2 and Figure 4). Predawn water potential has been reported as a reliable tree water status indicator for vines [23,35], while midday stem water potential is the reference tree water status indicator for stone fruit trees [8,36,37] and pome trees [38,39,40]. In our experiment with pear trees, recovery of Ψ_stem_ was observed during the early afternoon in fully irrigated trees, but trees with DI applied continued to decrease. The daily minimum values occurred during the early afternoon, mainly on those dates in July and August when the VPD reached the highest daily value (Figure 2). These results contrasted with the general belief that midday water potential reflects the most demanding moment of the day [41] and suggested that measuring Ψ_stem_ in anisohydric plants when the VPD is maximum is needed in order to identify early water stress. On 31 July and 11 August, Ψ_stem_ of both treatments were significantly different for the entire day, even when VPD, air temperature, and solar radiation were lower on 31 July than on 11 August. The lower sensitivity of Ψ_stem_ during the morning and midday compared to the afternoon (Table 1) has been related as an effect of the daily pattern of environmental conditions such as VPD, air temperature, and light intensity. The rapid changes of the environmental conditions during those periods of the day, as well as during the late afternoon, have been reported in warm climates to have a great impact on tree water status and to make measurements less reliable [42].

With respect to both methods compared, Ψ_stem_ values obtained using MT resulted in slightly higher variability compared to those measured with the PC (deviation that ranged 20%); however, it increased when the Ψ_stem_ decreased (Figure 4). Consequently, the greatest differences between measurements were observed when comparing minimum daily Ψ_stem_ values. These occurred during the afternoon and evening of days when evaporative demand was the highest. Minimum Ψ_stem_ values measured with MT were almost 0.6 MPa lower than those measured with PC when MT values were below −2.0 Mpa (Figure 6). The underestimation of Ψ_stem_ made by the MT could limit its use in those deficit irrigation strategies in which the Ψ_stem_ threshold value selected to irrigate the trees is −2.0 Mpa. Ψ_stem_ values below −2.0 Mpa have been broadly reported in experiments with olive trees [43], citrus [44], and almond trees [36] under deficit irrigation strategies. On the other hand, in fruit trees such as pears, apples, cherries, peaches, and nectarines, Ψ_stem_ values below −2.0 MPa are considered severe water stress and are undesirable as they will have a negative effect on current year fruit quality or yield or fruit quality the next year [45]. In pear trees, values below −1.1 MPa indicate water deficit conditions [46], values below −2.8 MPa induce cropping deficiencies the next year [47], and −3.5 MPa was suggested as the threshold value for vascular embolisms to develop [48]. Ψ_stem_ values reported here (Figure 2) were more negative than those reported in deficit irrigation experiments in pear trees under tropical climates [49] but similar to those reported under Mediterranean [50] and Oceanic climates [51]. The days selected to evaluate Ψ_stem_ measured using both MT and PC represented a wide range of conditions for the water status of trees. The difference between both methods was apparent when compared against VPD values (Figure 5). There was a stronger linear relationship between VPD and Ψ_stem_ measured using MT than between VPD and Ψ_stem_ measured using PC. The greatest dependency of Ψ_stem_ measured using MT on evaporative demand implies higher data variability and limits the contributions of the soil water availability in the assessment of the tree water status [52]. This situation was particularly clear during the evening when tree water status started to recover. There was an observed mismatch between Ψ_stem_ from MT which showed a later recovery than Ψ_stem_ from PC (Figure 6). In those measurements taken during the morning, this behavior was not observed.

When Ψ_stem_ of CTL and DI trees was compared, it was noticed that the differences in Ψ_stem_ between treatments were well differentiated and consistent for both methods (Figure 2). These results emphasize that although MT had greater variability and reported lower minimum values than those obtained with the PC, it can accurately assess tree water status within the range from −0.2 to −2.1 MPa. This covers the most commonly observed ranges in stem water potentials for most fruit trees [45]. Both methods had a similar signal intensity with values that reached 1.5 on 11 August. These values were similar to those reported for Ψ_stem_ in nectarine, apple, and pomegranate trees [37,38,53]. However, PC emerged as the method with the highest S due to a slightly lower CV (Table 1 and Table 2). The consistently higher CV of MT might be due to the expected differences found among trees within the same orchard (similar to 10%), but we hypothesize that the installation process might also be a source of variability. MT must be embedded in the trunk of the tree, so a wrong or loose contact between the sensor and the trunk may lead to unstable and highly variable measures and will increase the CV. Regarding the PC, as it is a manual measurement, the main source of variability is the effect of the operator on the Ψ_stem_ determination [54]. In this experiment and according to the analyzed data, we can state that both Ψ_stem_ methods are highly sensitive, strongly related with other plant water status indicators such as *G_s_* and T_c_-T_a_ and accurate to assess tree water status (Table 2; Figure 8 and Figure 9). Thus, when we compared the distribution of the values measured by each method (Figure 6), we observed that although the MT led to underestimating the minimum values and lightly overestimating the maximum values, and the Ψ_stem_ measured with them had greater dispersion, when those values were classified into a water stress scale, the differences between methods decreased, and both PC and MT showed the same distribution pattern (Figure 6). These results suggest that continuous monitoring of Ψ_stem_ is possible using MT in automated irrigation systems or as a useful tool to irrigation scheduling in fruit trees.

## 4. Materials and Methods

### 4.1. Study Site and Irrigation Treatments

The experiment was conducted during the 2021 growing season at the experimental orchard of the Washington State University located on Rock Island (Washington State, USA, 47°19′ N, 120°04′ W) on a 0.81 ha pear block (*Pyrus communis* L.), planted in 2007 on a shallow sandy loam soil. “D’Anjou” pear trees were grafted on OHxF.87 rootstock and trained on a central leader system at a tree density of 833 trees per hectare. Horticultural practices (e.g., fertilization, pruning, and weed control) were the same for all trees in the block and followed commercial regular practices. Full bloom was in April, and harvest was in late August. Trees were drip irrigated by a system consisting of a single drip line per tree row and five emitters per tree of 2 L h^−1^ discharge rate.

Two irrigation treatments were imposed: (1) A control treatment (CTL) irrigated at 100% of crop evapotranspiration (ET_c_) to ensure non-limiting soil water conditions, and (2) a regulated deficit irrigation treatment (DI), irrigated at 100% of ET_c_ from 1 April to 27 June, and 50% of ET_c_ from 28 June to 15 October. ET_c_ was calculated using the methodology proposed by Allen et al. [55]: ET_c_ = ET_0_ × K_c_ × K_r_, where ET_0_ is the reference evapotranspiration, K_c_ is the crop-specific coefficient reported for adult pear trees [46], and K_r_ is a factor of localization [56].

Treatments were arranged in a completely randomized block design with three replicates per treatment with six trees in each replicate. Two trees were selected for their uniformity (average ground cover of 41% and mean trunk diameter of 10.5 ± 0.23 cm) within each replicate for measurements.

### 4.2. Measurements

Four representative days with different atmospheric water demand, air temperature, and solar radiation were selected to evaluate tree water status under a wide range of environmental conditions: (i) a sunny, warm day with low evaporative demand (12 June 2021, which was before DI was initiated); (ii) a hot, sunny day with high evaporative demand (2 July 2021; five days after DI was initiated); (iii) a hot, cloudy day with high evaporative demand (31 July 2021; 35 days after DI was initiated); (iv) a hot, sunny day with high evaporative demand (11 August 2021; 46 days after DI was initiated).

#### 4.2.1. Environmental Data and Soil Water Content

Air temperature, relative humidity, wind speed, precipitation, solar radiation, and reference evapotranspiration were continuously recorded by an AgWeatherNet station located at the experimental orchard (http://www.weather.wsu.edu; 12 August 2021; “Sunrise station”). Two temperature and relative humidity sensors (ATMOS-14, METER Group Inc., Pullman, WA, USA) were also installed in the pear block. Mean air vapor pressure deficit (VPD) was calculated every 15 min using air temperature and relative humidity [52]. Soil volumetric water content (SWC) was obtained with two capacitance/frequency domain sensors (TEROS 11, Meter Group, Pullman, WA, USA) per replicate placed 25 and 50 cm below the surface and 25 cm from the drip emitter.

#### 4.2.2. Gas Exchange and Canopy Temperature

Leaf net photosynthesis (*P_n_*) and stomatal conductance (*G_s_*) were measured in six sun-exposed mature leaves from the outer part of the canopy per treatment. Measurements were made at 8:00, 10:30, 12:30, 15:30, 17:30, and 20:00 h using a portable gas exchange system (LI-6400, Li-Cor Inc., Lincoln, NE, USA) equipped with a 2 cm^2^ chamber at CO_2_ concentration of 400 µmol CO_2_ mol^−1^ air; the airflow rate inside the chamber was about 400 µmol s^−1^ and at environmental light and temperature conditions. The chamber incorporates a quantum sensor, and the photosynthetically photon flux density (PPFD) of the photosynthetic active radiation (PAR) was measured at every *P_n_* measurement. The canopy temperature (T_c_) was measured at the same time and in the same trees as gas exchange with a compact thermal camera (FLIR C2, FLIR Systems, Wilsonville, OR, USA). Images were taken 1.5 m from the sunny side of the canopy and were analyzed according to Blaya-Ros et al. [25]. The difference between the canopy and air temperature (T_c_-T_a_) was calculated.

#### 4.2.3. Stem Water Potential

**Figure 10:**
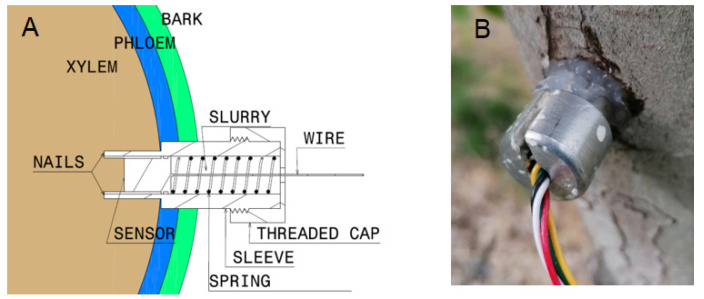
Microtensiometer installation scheme (**A**) and real sensor installed into the tree (**B**).

MT (FloraPulse, Davis, CA, USA) were embedded into the tree trunk of six trees for each DI and CTL treatment. The sensors were positioned on the North side of the trunk away from the sunlight. Figure 10 shows the MT installation scheme and the final result in the tree. Ψ_stem_ measurements with MT were recorded every 20 min using a solar-powered data logger (FloraPulse, Davis, CA, USA). Ψ_stem_ was also measured by using the Scholander pressure chamber (PC) in the same 6 trees at 6:00, 8:00, 10:00, 12:00, 14:00, 16:00, 18:00, and 20:00 h. Ψ_stem_ was measured with the PC (Model 615D, PMS Instrument Company, Albany, OR, USA) following methods described by McCutchan and Shackel [7]. Mature and healthy leaves close to the trunk were wrapped with black polyethylene bags and aluminum foil 2 h prior to the measurement. Measures were made on one leaf from each of the six trees per treatment in which the MT were installed.

Ψ_stem_ values were classified in a scale from 0 to 5 according to the tree water status, from the absence of water stress (0) to severe water stress (5): 0 for those values higher than −0.4 MPa, 1 between −0.4 and −0.7 MPa, 2 between −0.7 and −1.0 MPa, 3 between −1.0 and −1.3 MPa, 4 between −1.3 and −1.6 MPa, and 5 for lower Ψ_stem_ values than −1.6 MPa.

### 4.3. Sensitivity and Statistical Analysis

Sensitivity (S) was calculated according to Goldhamer and Fereres [57] for Ψ_stem_ measured with PC and MT, *G_s_*, and T_c_-T_a_. S was calculated dividing Signal Intensity (SI), calculated as the ratio of Ψ_stem_ between CTL and DI, by the coefficient of variation (CV). Data were analyzed by using analysis of variance (ANOVA) with a significance level of *p* < 0.05 (IBM SPSS Statistics, SPSS Inc., 24.0 Statistical package, Chicago, IL, USA). Linear regression analysis comparing Ψ_stem_ between PC and MT was performed with SigmaPlot 12.5 (Systat Software Inc., San Jose, CA, USA) and RStudio (RStudio Inc., Boston, MA, USA).

## 5. Conclusions

Microtensiometers provided continuous and accurate information of the tree water status. Ψ_stem_ values measured by the MT were strongly related to those measured with the PC across a range in Ψ_stem_ normally observed for irrigated woody plants. Both methods were highly sensitive and distinguished between different irrigation treatments. However, Ψ_stem_ values measured using MT had greater variability than Ψ_stem_ measured using a PC, and Ψ_stem_ values below −1.5 MPa were approximately 0.4 MPa lower for MT than PC. Plant water status indicators studied (*P_n_*, *G_s_*, T_c_ and Ψ_stem_) responded differently to the environmental forcing and to different irrigation strategies and resulted in being influenced by both daily and seasonal patterns. Maximum *P*_n_ was observed in the morning while those for *G_s_* and T_c_ were recorded at midday. On the other hand, Ψ_stem_ measured with both the PC and the MT showed the minimum daily values in the afternoon, when the VPD reached the highest daily values. Consequently, we propose measuring the gas exchange in the morning before the stomata closure, and the Ψ_stem_ when the maximum evaporative demand is recorded. Thus, according to our results, we conclude that MT represent an accurate continuous method for measuring Ψ_stem_ in trees during the growing season across a large range of environmental conditions and soil water content. Future works would be focused on using microtensiometers in automating irrigation systems and assessing if the reference lines and threshold values already proposed for Ψ_stem_ measured with the pressure chamber, especially for those trees highly tolerant to drought, could be used or should be adapted to this new and promising method.

## Figures and Tables

**Table 1:** Sensitivity analysis of stem water potential (Ψ_stem_) measured with the pressure chamber (PC) and the microtensiometers (MT), stomatal conductance (*G_s_*), and canopy-to-air temperature (T_c_-T_a_) for four periods throughout the day (N = 24).

	**Morning**				**Midday**			
	**Ψ_stem_**		***G_s_***	**T_c_-T_a_**	**Ψ_stem_**		***G_s_***	**T_c_-T_a_**
	**PC**	**MT**			**PC**	**MT**		
**SI ^1^**	1.26	1.30	0.90	0.71	1.30	1.26	0.83	0.45
**CV**	0.06	0.09	0.10	0.68	0.07	0.07	0.13	0.45
**S**	19.48	14.44	9.06	1.05	17.65	18.16	6.53	1.02
	**Afternoon**				**Evening**			
	**Ψ_stem_**		***G_s_***	**T_c_-T_a_**	**Ψ_stem_**		***G_s_***	T_c_-T_a_
	**PC**	**MT**			**PC**	**MT**		
**SI ^1^**	1.31	1.26	0.82	0.62	1.23	1.23	1.08	0.72
**CV**	0.04	0.07	0.12	0.37	0.04	0.05	0.20	0.30
**S**	34.02	18.16	7.00	1.67	29.93	23.89	5.52	2.42

^1^ SI: signal intensity (DI CTL^−1^); CV: coefficient of variation; S: sensitivity (SI CV^−1^).

**Table 2:** Sensitivity analysis of stem water potential (Ψ_stem_) measured with the pressure chamber (PC) and the microtensiometers (MT), stomatal conductance (*G_s_*), and canopy-to-air temperature (T_c_-T_a_) for different days throughout the growing season (N = 24).

	**12 June**				**2 July**			
	**Ψ_stem_**		***G_s_***	**T_c_-T_a_**	**Ψ_stem_**		***G_s_***	**T_c_-T_a_**
	**PC**	**MT**			**PC**	**MT**		
**SI ^1^**	1.03	1.08	1.01	0.98	1.09	1.09	0.96	0.66
**CV**	0.06	0.08	0.13	0.39	0.04	0.05	0.15	0.73
**S**	17.06	13.81	8.03	2.53	27.60	21.14	6.55	0.91
	**31 July**				**11 August**			
	**Ψ_stem_**		***G_s_***	**T_c_-T_a_**	**Ψ_stem_**		***G_s_***	**T_c_-T_a_**
	**PC**	**MT**			**PC**	**MT**		
**SI ^1^**	1.43	1.36	0.79	0.31	1.54	1.52	0.87	0.56
**CV**	0.06	0.08	0.11	0.41	0.05	0.07	0.16	0.27
**S**	22.64	16.47	7.24	0.75	28.30	22.31	5.51	2.08

^1^ SI: signal intensity (DI CTL^−1^); CV: coefficient of variation; S: sensitivity (SI CV^−1^).
